# Microarray Analysis Reveals Overexpression of both Integral Membrane and Cytosolic Tight Junction Genes in Endometrial Cancer Cell Lines

**DOI:** 10.7150/jca.75510

**Published:** 2022-10-31

**Authors:** Maria E Cuevas, Chance P Winters, Maria C Todd

**Affiliations:** Biology Department, Southwestern University, Georgetown, TX 78626.

**Keywords:** endometrial cancer, claudin-3, claudin-4, F11R, TPJ3, microarray

## Abstract

Deregulation of tight junction (TJ) proteins and the associated disruption of TJ function has been demonstrated to play a role in the development of endometrial cancer. In the current study, we have shown overexpression of claudin-3 and -4 mRNA (by RT-PCR) and protein (by immunoblotting) in a panel of 9 human endometrial cancer cell lines. To further expand our understanding of the complex role of TJ deregulation in endometrial cancer, we also investigated the expression of 84 TJ and TJ-associated genes (encoding the array of proteins that function within the TJ network from the membrane to nuclear signaling pathways) by microarray analysis. Consistent with the claudin-3 and -4 RT-PCR and immunoblot findings described above, we observed overexpression of the claudin-3 and -4 genes by microarray analysis. Further, we observed overexpression of an additional three genes in 8 of the 9 endometrial cancer cell lines: OCLN (occludin), F11R (JAM-A) and TJP3 (ZO-3). OCLN and F11R encode integral membrane proteins whereas TJP3 encodes a cytosolic scaffolding protein that indirectly links membrane TJ proteins to the actin cytoskeleton and cell signaling pathways. Our data suggest that the structural disruption of TJs coupled with the downstream deregulation of signaling pathways involved in cellular proliferation and migration may contribute to the development of endometrial cancer.

## Introduction

Endometrial cancer is the most common female reproductive cancer in the United States and so far has accounted for an estimated 12,550 deaths in 2022 1. Endometrioid adenocarcinomas are the most common type of endometrial cancer, accounting for approximately 75% of all cases. Less common and more aggressive endometrial cancer histopathologic subtypes include clear-cell carcinoma, mucinous adenocarcinoma and papillary serous adenocarcinoma 2-3.

Over the last few years, we and others have demonstrated a role for tight junction (TJ) deregulation in the development of endometrial cancer 4 (for a review see 5). TJs, which consist of a complex of integral membrane and associated cytoplasmic proteins, play major roles in cell-to-cell adhesion, regulation of paracellular transport and maintenance of cell polarity (for a review see 6). In addition, studies have also suggested a role for TJ proteins in recruiting signaling proteins that regulate such processes as gene transcription, cellular division and specialization 7-9. Given the many and diverse roles of TJs it is likely that the loss of TJ integrity may contribute to cancer development and progression.

The TJ protein complex consists of the integral membrane proteins: claudins (CLDNs), occludin (OCLN), junctional adhesion molecules (JAMs) and the coxsackievirus and adenovirus receptor (CAR). Investigations into the role of TJ deregulation in endometrial cancer have primarily focused on the expression of CLDNs and OCLN. Indeed, several studies have shown overexpression of CLDNs-3 and -4 in uterine serous papillary carcinoma 10-11, clear-cell endometrial carcinoma 10 and uterine carcinosarcoma 12. Notably, overexpression of CLDNs-3 and -4 was associated with a poor clinical outcome 11. Endometrioid adenocarcinomas expressing particularly high levels of CLDN-3 and -4 proteins have been found to exhibit structurally disrupted TJs 13. Consistent with these findings, overexpression of these two CLDN proteins has been positively correlated with tumor progression in the endometrium and increased myometrial invasion 13. CAR expression has also been found to increase with tumor grade in endometrial cancer 14. Whereas, the vast majority of studies have shown overexpression of CLDNs 3 and -4, levels of OCLN and JAM-A TJ membrane proteins have been found to be decreased in endometrial cancer 15-16. TJ protein networks also include a number of cytosolic adapter proteins known as zona occludens (ZO-1, ZO-2 and ZO-3) that are associated with the intracellular domains of the integral membrane proteins described above. These adapter proteins play a crucial bridging role in linking TJs to the cytoskeleton and signaling pathways 9. Because there are only very few studies that address the deregulation of these cytosolic proteins in endometrial cancer, we are currently lacking a complete understanding of the contribution of TJ deregulation to endometrial cancer.

In the current study we have demonstrated the deregulated expression of CLDNs-3 and -4 in a panel of human endometrial cancer cell lines. In addition, to gain a more global understanding of the role of TJ deregulation in endometrial cancer development we have evaluated those same cell lines for the expression of 84 TJ and TJ-associated genes through microarray analysis.

Our study found that all but one of the cell lines showed overexpression of an additional three TJ genes: OCLN, F11R (JAM-A) and TJP3 (ZO-3), suggesting a more complex role for TJ deregulation in endometrial cancer involving proteins that function at different levels within the TJ network from cell to cell adhesion to cell signaling.

## Materials and methods

### Cell lines and culture conditions

The normal endometrial cell line THESCs (CRL-4003) and the endometrial cancer cell lines, AN3-CA (HTB-111), KLE (CRL-1622), MES-SA (CRL-1976), RL95-2 (CRL-1671), and SK-UT-1 (HTB-114) were obtained from the American Type Culture Collection (ATCC, Manasas, VA, USA). The endometrial cancer cell lines EFE-184 (ACC-230), MFE-280 (ACC-410), MFE-296 (ACC 419) and MFE-319 (ACC 423) were purchased from Deutsche Sammlung von Mikroorganismen und Zellkulturen DSMZ (Braunschweig, Germany). The cancer cell lines were cultured in their respective medium as stipulated by ATCC or DSMZ supplemented with 10-20% fetal bovine serum (FBS) (ThermoFisher Scientific, Carlsbad, CA, USA; cat # 10438026), 1% penicillin/streptomycin/2mM-glutamine (ThermoFisher, Waltham, MA, USA; cat # 10378106). The normal endometrial cell line (THESCs) was cultured in phenol-free medium per ATCC instructions supplemented with 1.5 g/l sodium bicarbonate, 1% ITS+ Premix (BD, San Jose, CA, USA; cat # 354352), 500ng/ml puromycin, 10% charcoal/dextran treated fetal bovine serum (CSFBS) (Hyclone, Logan, UT, USA; cat # SH30068.03). All cell lines were maintained in a 5% CO_2_ humidified atmosphere at 37^o^C.

### Protein extraction

Cells were harvested by washing with PBS, followed by treatment with trypsin/EDTA (0.25%/1mM). Protein extracts were obtained by resuspending the cell pellet in Laemmli sample buffer/5% β-mercaptoethanol (BioRad, Hercules, CA, USA; cat # 1610737). The extracts were boiled at 100^o^C for 3 min, sheared and stored at -80°C.

### RNA extraction

RNA was isolated from endometrial cells (1 x 10^6^ cells/ml) using the Qiagen RNeasy Mini Kit (Hilden, Germany; cat # 74104) according to the manufacturer's instructions. RNA extracts were stored at -80°C.

### Immunoblot analysis

Approximately 30 µg of each of the protein extracts was subjected to electrophoresis on precast 10% SDS-polyacrylamide gels (BioRad, Amersham, Buckinghamshire, UK; cat # 456-1033) and electrophoretically transferred to Immobilon-P PVDF membranes (Merck Millipore Ltd, Co. Cork, Ireland; cat # IPVH304F0). The membranes were probed for 1 h at room temperature with 2 μg/ml rabbit polyclonal anti-human claudin-3 (Life Technologies, Carlsbad, CA, USA; cat # 34-1700) or 3 μg/ml mouse monoclonal anti-human claudin-4 (Life Technologies, cat # 32-9400) primary antibodies in 5% milk/PBS solution. The membranes were washed in PBS for 10 min, PBS/0.1% TWEEN for 5 min (twice) then PBS for 10 min. The membranes were then incubated with 1:3000 HRP-conjugated goat anti-rabbit (BioRad; cat # 403005) or goat anti-mouse (BioRad; cat # 5178-2504) secondary antibodies for 1 h at room temperature then washed as described above. Membranes were incubated with the enhanced chemiluminescence ECL^™^-plus kit (VWR, Radnor, PA, USA; cat # 89168-782) according to the manufacturer's instructions, then the signals were visualized using the Invitrogen™ iBright™ FL1500 (Thermo Fisher Scientific, Waltham, MA, USA). As a loading control, membranes were re-probed with 4.0µg/ml mouse monoclonal anti-human GAPDH (Ambion, Life Technologies; cat # AM4300).

### RT-PCR analysis

Prior to reverse transcription, RNA samples were treated with DNase I (Invitrogen, Waltham, MA, USA; cat # 18068-015). RNA was then reversed transcribed into cDNA using the Q-OMNISCRIPT^®^ RT-PCR kit according to the manufacturer's instructions (Qiagen, Germantown, MD, USA; cat # 205113). Briefly, a 20 μl reaction contained 2 μg of RNA, 1X Buffer RT, 5 mM dNTPs, 10 U/ml of RNase inhibitor, and 1 U/ml of reverse transcriptase. The SYBR Green I assay and Applied Biosystems StepOnePlus^TM^ were used to detect RT-PCR products (Applied Biosystems, Foster City, CA, USA). Each 20 μl RT- PCR reaction contained 0.1 μg of cDNA; 400 nM each of the following primer sequences taken from Pan et al. (2007 13: CLDN-3 (forward, 5'-ctgctctgctgctcgtgtcc-3'; reverse, 5'-ttagacgtagtccttgcggtcgtag-3'); CLDN-4 (forward, 5'-gtgccttgctcaccgaaac-3'; reverse, 5'-ccaccactgcccaaacct-3'); and glyceraldehyde phosphate dehydrogenase (GAPDH) (forward, 5' -gaagatggtgatgggatttc-3'; reverse, 5' -gaaggtgaaggtcggagt-3'), 10 μl PCR master mix and 0.16 μl RT SYBR Green mix. The cycling conditions used were previously described by Pan et al. (2007) 13. Briefly, denaturation for 10 min at 95^o^C followed by 40 cycles of amplification and quantification (10 sec at 95^o^C; 5 sec at 57^o^C; 10 sec at 72^o^C with a single fluorescence measurement). The melting curve was measured at 65-95^o^C with a heating rate of 0.1^o^C per sec and a continuous fluorescence measurement. Each reaction was performed in triplicate. The relative expression was calculated using the 2 ^-△△Ct^ method (quantity of target, normalized to GAPDH endogenous control and relative to a reference sample, THESCs). Statistical significance was evaluated for each target gene separately using one-way ANOVA and post-hoc Tukey HSD test compared to THESCs (n = 4); *p<0.05; ** p<0.01 was considered to indicate a statistically significant result.

### Microarray analysis

Using a SYBR green-based quantitative real-time PCR Human Tight Junctions RT^2^ Profiler PCR Array (Qiagen; cat # PAH5.1432C-2) and the Applied Biosystems StepOnePlus^TM^ (Applied Biosystems), the expression of 84 tight junction genes was determined. In addition to the tight junction genes, the 96-well RT^2^ profiler microplate contained primers for 5 housekeeping genes, one DNA contamination control, three reverse transcription controls and three positive PCR controls. Briefly, the RNA from endometrial cancer cell lines and the normal endometrial cell line (THESCs) was isolated using the RNeasy Mini Kit (Qiagen; cat # 74104) according to the manufacturer's instructions. RNA samples were reverse transcribed into cDNA using the RT^2^ first strand kit (Qiagen, cat # 330401). cDNA was mixed with the RT^2^ SYBR Green mastermix (Qiagen, cat # 330500) and 25 μl of the PCR mix was aliquoted into each well of the RT^2^ profiler PCR array microplate. The PCR program consisted of an initial denaturation period (10 min at 95^o^C) followed by 40 cycles of amplification and quantification (15 sec at 95^o^C; 1 min 60^o^C). The relative expression was based on the expression ratio of each of the TJ genes and was determined using the 2 ^-△△Ct^ method as described above (RT^2^ Profiler Array Data Analysis WebPortal).

## Results

### Overexpression of claudin-3 and -4 proteins in endometrial cancer cell lines by immunoblot analysis

**Figure 1:**
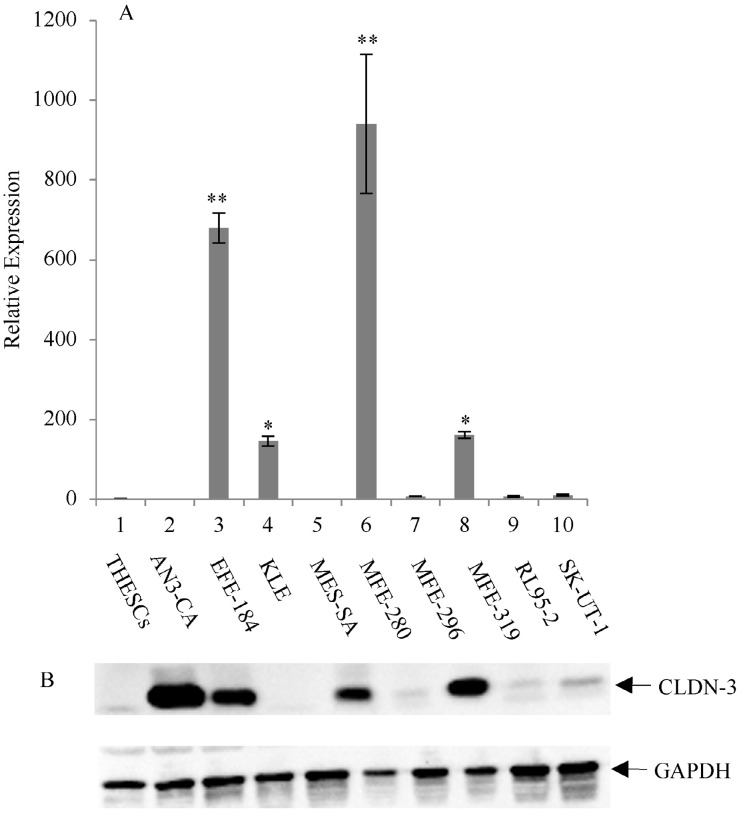
** Overexpression of CLDN-3 in a panel of endometrial cancer cell lines.** (A) Real-time quantitative PCR analysis of CLDN-3 gene expression. RNA isolated from a panel of 9 endometrial cancer cell lines and one normal endometrial cell line (THESCs) was amplified using primers designed to the CLDN-3 and GAPDH (control) genes. Values are mean + SD (n=4). Statistical significance was evaluated for each target gene separately using one-way ANOVA and post-hoc Tukey HSD test compared to THESCs; *p<0.05; ** p<0.01. (B) Immunoblot analysis of CLDN-3 protein expression. The same panel of normal and tumor endometrial cell lines was subjected to immunoblot analysis using antibodies to CLDN-3 and GAPDH (loading control).

**Figure 2:**
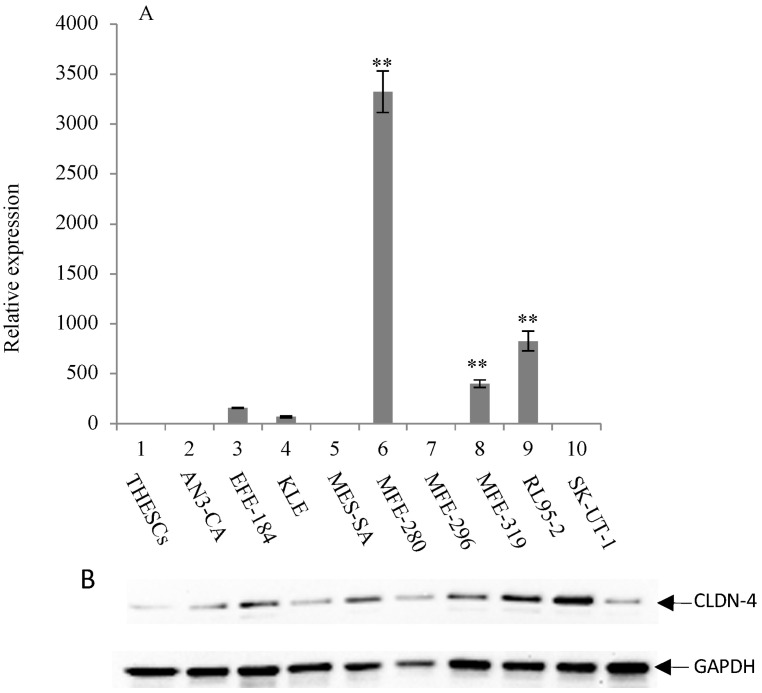
** Overexpression of CLDN-4 in a panel of endometrial cancer cell lines**. (A) Real-time quantitative PCR analysis of CLDN-4 gene expression. RNA isolated from a panel of 9 endometrial cancer cell lines and one normal endometrial cell line (THESCs) was amplified using primers designed to the CLDN-4 and GAPDH (control) genes. Values are mean + SD (n=4). Statistical significance was evaluated for each target gene separately using one-way ANOVA and post-hoc Tukey HSD test compared to THESCs; *p<0.05; ** p<0.01. (B) Immunoblot analysis of CLDN-4 protein expression. The same panel of normal and tumor endometrial cell lines was subjected to immunoblot analysis using antibodies to CLDN-4 and GAPDH (loading control).

The expression of CLDN-3 and -4 proteins in a panel of nine endometrial cancer cell lines was determined by immunoblot analysis (Figure 1B and 2B). The normal endometrial cell line, THESCs was included in the panel as a control. Whereas the THESCs cells expressed barely detectable levels of CLDN-3 and -4 proteins, we observed dramatic overexpression of CLDN-3 in AN3-CA, EFE-184, MFE-280 and MFE-319 endometrial cancer cell lines relative to THESCs (Figure 1B). In addition, although the overall levels of CLDN-4 protein were lower compared with those of CLDN-3, we also observed overexpression of CLDN-4 in the EFE-184, MES-SA, MFE-296, MFE-319 and RL95-2 endometrial cancer cell lines relative to THESCs (Figure 2B).

### Overexpression of claudin-3 and -4 mRNA in endometrial cancer cell lines by RT-PCR

To investigate the mechanism of CLDN-3 and -4 protein overexpression, we subjected RNA isolated from the same panel of endometrial cancer cell lines to RT-PCR analysis using primers targeted to the CLDN-3 and -4 genes (Figure 1A and 2A). We observed reasonably good agreement between the levels of CLDN mRNA and protein in the panel of cell lines. In particular we observed a consistent pattern of CLDN-3 protein and mRNA overexpression in three cell lines: EFE-184, MFE-280 and MFE-319 (Figure 1A/B). Similarly, there was a consistent pattern of CLDN-4 protein and mRNA overexpression in four cell lines: EFE-184, MFE-280, MFE-319 and RL95-2 (Figure 2A/B). Whereas the pattern of protein and mRNA expression was consistent in the majority of the cell lines, there were notable exceptions. Specifically, AN3-CA showed overexpression of CLDN-3 protein but no overexpression of mRNA relative to THESCs (Figure 1A/B). Conversely, MFE-280 showed overexpression of CLDN-4 mRNA but no overexpression of protein relative to THESCs (Figure 2 A/B).

### Overexpression of integral membrane tight junction and cytosolic tight junction-associated genes revealed by microarray analysis

Following the expression analyses of CLDN-3 and -4, we expanded our study to simultaneously analyze the expression of 84 TJ and TJ-associated genes in the same 9 endometrial cancer cell lines (described above) using the RT^2^ Profiler PCR microarray (Table 1). As expected, both RT-PCR and microarray analyses revealed similar trends in the expression of the CLDN-3 and -4 genes. Of the 84 genes in the microarray, 24 were overexpressed in at least one of the endometrial cancer cell lines relative to the normal cell line, THESCs (Table 1). In addition, 8 of the 9 cell lines showed overexpression (relative to THESCs) of the three tight junction genes: OCLN (occludin) (which ranged in expression from 0.35-30-fold), F11R (JAM-A) (which ranged in expression from 7.5-1000-fold) and TJP3 (ZO-3) (which ranged in expression from 0.22-44.5-fold). Notably, the MFE-280 endometrial cancer cell line overexpressed 23 of the 24 genes that were found to be overexpressed in at least one each of the other 8 cell lines. The remaining genes in the microarray were expressed at the same level in each of the endometrial cancer cell lines as that expressed by the normal THESCs cell line.

## Discussion

In the current study, we investigated the contribution of deregulated TJ gene expression to endometrial cancer development. Using immunoblot analysis, we demonstrated overexpression of the CLDN-3 and -4 integral membrane proteins in 4 of 9 and 5 of 9 endometrial cancer cell lines, respectively. RT-PCR analysis of the same cell lines indicated increased mRNA transcription as the underlying mechanism for the protein overexpression. In the case of two cell lines, there were inconsistencies between the patterns of CLDN protein and mRNA expression. AN3-CA, showed overexpression of the CLDN-3 protein but no associated overexpression of the CLDN-3 mRNA. This finding could be due to stabilization of the CLDN-3 protein in this cell line. In contrast, the MFE-280 cell line showed overexpression of CLDN-4 mRNA but no associated protein overexpression. It is possible that defective RNA processing may be responsible for this discrepancy.

Consistent with our findings, multiple groups have shown deregulated expression (sometimes associated with mislocalization) of CLDN-3 and -4 integral membrane proteins 4, 10-13, 17. The resulting disruption of tight junctions and weakening of cell-to-cell adhesion 13, 18-19 may increase the access of tumor cells to nutrients and facilitate cell migration 13, 20-21. Indeed, overexpression of CLDN-3 and -4 is associated with progression of disease and poor clinical outcome in endometrial cancer patients 10-12.

To-date, much research has focused on the role of deregulated claudin and occludin proteins in cancer development. However, these integral membrane proteins constitute just one component of the complex network of TJ proteins that contribute to a wide and diverse array of cellular processes. To gain a greater understanding of the role of TJ deregulation in cancer development, we expanded our analysis beyond integral membrane proteins to investigate the expression of genes encoding TJ-associated proteins found in the cytosol and cytoskeleton. Using a TJ microarray, we were able to analyze the expression of 84 different TJ genes. In addition to the expected frequent overexpression of the much studied CLDN-3, CLDN-4 and OCLN genes, we also observed overexpression of an additional 21 TJ genes in at least one of the endometrial cancer cell lines. Notably, a further two genes - F11R (JAM-A) and TJP3 (ZO-3) were overexpressed in 8 of the 9 cell lines. These findings are consistent with a study by Colas et al. (2011) 21 who showed overexpression of TJP3 (ZO-3) mRNA in endometrial cancer tissues compared with normal endometrial tissues. In addition, a study by Koshiba et al., (2009) 16 showed overexpression of F11R (JAM-A) in well differentiated adenocarcinoma but reduced expression in less differentiated adenocarcinoma. F11R (JAM-A) is a single membrane-spanning TJ protein involved in the maintenance of cellular polarity and TJP3 (ZO-3) is a scaffolding protein that indirectly links membrane TJ proteins to the actin cytoskeleton and cell signaling pathways 9, 22. Given the functions of these proteins, deregulation of their expression is likely to affect both the overall structural integrity of TJs and the membrane to nuclear signaling pathways that regulate cellular proliferation and migration of endometrial cancer cells.

By studying the expression of all of the known TJ and TJ-associated genes in a panel of endometrial cancer cell lines, our microarray analysis indicates a more complex role for TJ deregulation in tumor etiology. Specifically, we have confirmed the role of deregulated CLDN and OCLN genes (which encode integral membrane proteins) and provided strong additional evidence for deregulated F11R (JAM-A) and TJP3 (ZO-3) in the development of endometrial cancer. These data provide potential gene candidates for the development both of diagnostic markers and novel gene-based therapeutic approaches to the treatment of endometrial cancer.

## Figures and Tables

**Table 1:** Differential expression of tight junction genes in a panel of endometrial cancer cell lines

			Fold increase relative to normal cell line THESCs								
Gene	Name	Accession No.	AN3-CA	EFE 184	KLE	MES-SA	MFE280	MFE296	MFE 319	RL95-2	SK-UT-1
ACTN2	Actin-Alpha-2	NM_001102		2.4		2.6	34.3				7.0
CLDN-3	Claudin-3	NM_001306	3.7	3.1 x 10^2^	12	4.0	4.6 x 10^3^	4.9	5.1 x 10^2^	30.27	41.5
CLDN-4	Claudin-4	NM_001305		2.6	2.0		4.1 x 10^2^		68.5	1.5 x 10^3^	0.43
F11R	F11 Receptor	NM_016946		59	65.9	7.5	1.0 x 10^3^	27.79	3.9 x 10^2^	7.3 x 10^2^	13.5
TJP3	Tight junction protein 3 (ZO-3)	NM_014428		1.5	2.1	1.0	29.6	0.35	30.8	44.5	0.22
CLDN-1	Claudin-1	NM_021101		6.3	1.8		1.1 x 10^2^				
CLDN-5	Claudin-5	NM_003277					3.6				
CLDN-6	Claudin-6	NM_021195					29.3				
CLDN-7	Claudin-7	NM_001307		2.3	42.6		90.3		31.9		
CLDN-8	Claudin-8	NM_199328	7.0		2.2 x10^2^	17.2	2.7 x 10^2^	1.5			3.2
CLDN-16	Claudin-16	NM_006580		1.7 x 10^2^	0.9		60.6	216			
OCLN	occludin	NM_002538	0.01	0.6	4.3	0.08	30.4	0.4	0.48	2.9	0.35
LLGL2	Lethal giant larvae homolog 2 (Drosophila)	NM_004524	6.4			2.5	60.5	2.9	47.4	43.5	1.7
TIAM1	cell lymphoma invasion and metastasis 1	NM_003253					41.1				
ESAM	Endothelial cell adhesion molecule	NM_138961					41.0				
CGN	Cingulin	NM_020770		6.0	5.9		68.2			30.6	
EPB41	Erythrocyte membrane protein band 4.1 (eliptocytosis 1, RH linked)	NM_004437					2.9				
INADL	InaD-like (Drosophila)	NM_176877					4.6				
MLLT4	Myeloid/lymphoid or mixed lineage leukemia (trithorax homolog, Drosophila) translocated to, 4	NM_001040000					2.2				
PARD6A	Par-6-partitioning defective 6 homolog alpha (C elegans)	NM_016948			8.1		11.4				
PARD6B	Par-6-partitioning defective 6 homolog beta (C elegans)	NM_032521	2.1		11.6	1.3	29.2				
PECAM1	Platelet/endothelial cell adhesion molecule	NM_000442	4.1			3.6	5.7				4.6
PRKCI	Protein Kinase C, iota	NM_002740			11.6		2.0				

## References

[2] Setiawan VW, Yang HP, Pike MC, McCann SE, Yu H, Xiang YB, Wolk A, Wentzensen N, Weiss NS, Webb PM, Van Den Brandt PA (2013). Type I and II endometrial cancers: have they different risk factors?. J Clin Oncol.

[3] Brinton L, Felix AS, McMeekin DS, Creasman WT, Sherman ME, Mutch D, Cohn DE, Walker L, Moore RG, Downs LS, Soslow RA (2013). Etiologic heterogeneity in endometrial cancer: evidence from a gynecologic oncology group trial. Gynecol Oncol.

[4] Cuevas ME, Gaska JM, Gist AC, King JM, Sheller RA, Todd MC (2015). Estrogen-dependent expression and subcellular localization of the tight junction protein claudin-4 in the endometrial HEC-1A cancer cells. Int J Oncol.

[5] Leech AO, Cruz RGB, Hill ADK, Hopkins AM (2015). Paradigms lost: an emerging role for over-expression of tight junction adhesion proteins in cancer pathogenesis. Ann Trans Med.

[6] Zihni C, Mills C, Matter K, Balda M (2016). Tight Junctions: from simple barriers to multifunctional molecular gates. Nature Review Molec Cell Biol.

[7] Turksen K, Troy TC (2004). Barriers built on claudins. J Cell Sci.

[8] Goetsch L, Haeuw JF, Beau-Larvor C, Gonzalez A, Zanna L, Malissard M, Lepecquet AM, Robert A, Bailly C, Broussas M, Corvaia N (2013). A novel role for junctional adhesion molecule-A in tumor proliferation: modulation by an anti-JAM-A monoclonal antibody. Int J Cancer.

[9] Gonzalez-Marisca L, Miranda J, Ortega-Olvera J, Gallego-Gutierrez H, Raya-Sandino A, Vargas-Sierra O (2016). Zonula Occludens Proteins in Cancer. Curr Pathobiol Rep.

[10] Santin AD, Bellone S, Marizzoni M, Palmieri M, Siegel ER, McKenney JK, Hennings L, Comper F, Bandiera E, Pecorelli S (2007). Overexpression of claudin-3 and claudin-4 receptors in uterine serous papillary carcinoma: novel targets for a type specific therapy using Clostridium perfringens enterotoxin (CPE). Cancer.

[11] Konecny GE, Agarwal R, Keeney GA, Winterhoff B, Jones MB, Mariani A, Riehle D, Neuper C, Dowdy SC, Wang HJ, Morin PJ, Podratz KC (2008). Claudin-3 and claudin-4 expression in serous papillary, clear-cell, and endometrioid endometrial cancer. Gyno Oncology.

[12] Gaetje R, Holtrich U, Engels K, Kissler S, Rody A, Karn T, Kaufmann M (2008). Differential expression of claudins in human endometrium and endometriosis. Gynecol Endocrinol.

[13] Pan XY, Wang B, Che YC, Weng ZP, Dai HY, Peng W (2007). Expression of claudin-3 and claudin-4 in normal, hyperplastic, and malignant endometrial tissue. Int J Gynecol Cancer.

[14] Giaginis C, Zarros A, Alexandrou P, Klijanienko J, Delladetsima I, Theocharis S (2010). Evaluation of coxsackievirus and adenovirus receptor expression in human benign and malignant thyroid lesions. APMIS.

[15] Tobioka H, Isomura H, Kokai Y, Tokunaga Y, Yamaguchi J, Sawada N (2004). Occludin expression decreases with the progression of human endometrial carcinoma. Hum Path.

[16] Koshiba H, Hosokawa K, Kubo A, Tokumitsu N, Watanabe A, Honjo H (2009). Junctional adhesion molecule A [corrected] expression in human endometrial carcinoma. Int J Gynecol Cancer.

[17] Corsini M, Ravaggi A, Odicino F, Santin AD, Ravelli C, Presta M, Romani C, Mitola S (2018). Claudin 3 is localized outside the tight junctions in human carcinomas. Oncotarget.

[18] Sheller RA, Cuevas ME, Todd MC (2017). Comparison of Transepithelial resistance measurements techniques: Chopsticks vs. Endhom. Biological Procedures Online.

[19] Todd MC, Petty HH, King JM, Piana-Marshall BN, Sheller RA, Cuevas ME (2015). Overexpression and delocalization of claudin-3 protein in MCF-7 and MDA-MB-415 breast cancer cell lines. Oncol Lett.

[20] Agarwal R, D'Souza T, Morin PJ (2005). Claudin-3 and claudin-4 expression in ovarian epithelial cells enhances invasion and is associated with increased matrix metalloproteinase-2 activity. Cancer Res.

[21] Colas E, Perez C, Cabrera S, Pedrola N, Mong M, Castellvi J, Eyzaguirre F, Gregorio J, Ruiz A, Llaurado M, Rigau M, Garcia M, Ertekin T, Montes M, Lopez-Lopez R, Carreras R, Xercavins J, Ortega A, Maes T, Rosell E, Doll A, Abal M, Reventos J, Gil-Moreno A (2011). Molecular markers of endometrial carcinoma detected in uterine aspirates. Int J Cancer.

[22] Balda M, Matter K (2009). Tight Junctions and the Regulation of Gene Expression. Biochim Biophys Acta.

